# Cultivation and Genomic Characterization of the Bile Bacterial Species From Cholecystitis Patients

**DOI:** 10.3389/fmicb.2021.739621

**Published:** 2021-11-01

**Authors:** Qiulong Yan, Siyi Zhang, Shenghui Li, Guangyang Wang, Aiqin Zhang, Taiyang Jin, Yue Zhang, Qingbo Lv, Manchun Xiao, Yuanyuan Sun, Xiang Li, Song Cui, Rui Li, Xiaochi Ma, Chao Wang, Xiangge Tian, Xiaohui Duan, Yi Xin, Xianhai Mao, Yufang Ma

**Affiliations:** ^1^Department of Microbiology, College of Basic Medical Sciences, Dalian Medical University, Dalian, China; ^2^Department of Hepatobiliary Surgery, Hunan Provincial People’s Hospital, The First Affiliated Hospital of Hunan Normal University, Changsha, China; ^3^Puensum Genetech Institute, Wuhan, China; ^4^Department of Biotechnology, College of Basic Medical Sciences, Dalian Medical University, Dalian, China; ^5^College of Pharmacy, Dalian Medical University, Dalian, China

**Keywords:** bile bacteria, genomic characterization, antibiotic resistance genes, virulence factors, culturome, cholecystitis patients

## Abstract

The microbes in human bile are closely related to gallbladder health and other potential disorders. Although the bile microbial community has been investigated by recent studies using amplicon or metagenomic sequencing technologies, the genomic information of the microbial species resident in bile is rarely reported. Herein, we isolated 138 bacterial colonies from the fresh bile specimens of four cholecystitis patients using a culturome approach and genomically characterized 35 non-redundant strains using whole-genome shotgun sequencing. The bile bacterial isolates spanned 3 classes, 6 orders, 10 families, and 14 genera, of which the members of *Enterococcus*, *Escherichia–Shigella*, *Lysinibacillus*, and *Enterobacter* frequently appeared. Genomic analysis identified three species, including *Providencia* sp. D135, *Psychrobacter* sp. D093, and *Vibrio* sp. D074, which are not represented in existing reference genome databases. Based on the genome data, the functional capacity between bile and gut isolates was compared. The bile strains encoded 5,488 KEGG orthologs, of which 4.9% were specific to the gut strains, including the enzymes involved in biofilm formation, two-component systems, and quorum-sensing pathways. A total of 472 antibiotic resistance genes (ARGs) were identified from the bile genomes including multidrug resistance proteins (42.6%), fluoroquinolone resistance proteins (12.3%), aminoglycoside resistance proteins (9.1%), and β-lactamase (7.2%). Moreover, *in vitro* experiments showed that some bile bacteria have the capabilities for bile salt deconjugation or biotransformation (of primary bile acids into secondary bile acids). Although the physiological or pathological significance of these bacteria needs further exploration, our works expanded knowledge about the genome, diversity, and function of human bile bacteria.

## Introduction

Cholecystitis leads to gallbladder inflammation or even perforation, tissue death, gangrene, fibrosis, and shrinking of the gallbladder, which is a hospitalized disease with increasing medical and financial burden (Wadhwa et al., 2017; Wang et al., 2021). The cardinal symptoms were fever, pain, and nausea with increasing levels of clinical indicators, such as serum transaminases, alkaline phosphatase, and total bilirubin (Chisholm et al., 2019). Laparoscopic cholecystectomy was commonly applied for treating this disease (Loozen et al., 2018).

Although the pathogenicity in cholecystitis still needs further exploration, bacteria, as one of the important risk factors, were closely correlated with its poor operative outcomes (Liu et al., 2015; Yun and Seo, 2018). Moreover, positive bile culture rates from laparoscopic cholecystectomy patients ranged from 25.1 to 60.3%, suggesting the presence of bacteria in the cholecystitis patients’ bile (Nitzan et al., 2017; Yun and Seo, 2018). Due to the limitation of culture conditions, most bile bacterial isolates from clinical samples are aerobic bacteria especially Gram stain-negative Enterobacteriaceae, such as *Escherichia coli*, *Klebsiella* spp., *Enterobacter* spp., and less frequently anaerobic bacteria, including *Bacteroides* and *Clostridium* spp., as well as microaerophilic *Helicobacter pylori* (Nitzan et al., 2017; Cen et al., 2018). For culture-independent methods, denaturing gradient gel electrophoresis (DGGE) was applied for the investigation of microbiota composition (Liu et al., 2015); however, low throughput and complex operation were its limitations. With the advent of high-throughput sequencing platforms and bioinformatic analysis pipelines, metagenome sequencing analysis including 16S rRNA amplicon sequencing and whole genomic shotgun sequencing have revealed the abundant diversity composition of the bile microbiome (Wu et al., 2013; Liu et al., 2015). Meanwhile, owing to nearly 80% of the human intestinal bacteria were unculturable (Tajeddin et al., 2016), with the advancement in bacterial culturomics technology (Tidjani Alou et al., 2020), it is reasonable to infer that abundant bile bacteria are waiting to be isolated.

Proper identification method of the isolated bacteria was a highly essential step in the culture-dependent workflow. The traditional polyphasic taxonomic strategy aims to generate phenotypic and phylogenetic information of a bacterium, such as its shape, color, size, staining properties, host range, pathogenicity, indole test, gelatin liquefaction test, motility test, and assimilation of carbon sources (Das et al., 2014). In addition to biochemical identification, flow cytometry and MALDI-TOF mass spectrometry can also be used for rapid identification for cultured isolates (Das et al., 2014; Jang and Kim, 2018). Despite each approach having its own advantages and limitations, such as higher cost or rough results of flow cytometer and equipment popularity of MALDI-TOF mass spectrum hindered their application. Considerable advancement in culturable bacterial identification and taxonomy methods using genotypic and phylogenetic information has taken place such as chemotaxonomy, numerical taxonomy, and DNA–DNA hybridization, housekeeping gene amplification, and sequencing (16S rRNA gene, *gyrB*, *rpoABCD*, etc.) and whole-genome sequencing (Das et al., 2014). As the primary gene target for bacterial identification and comprehensive consideration of cost performance, the 16S rRNA gene was often used for preliminary screening. Then whole-genome sequencing was used for in-depth exploration of functional genes. In recent years, cultivation-dependent microbiota studies of the human digestive tract (Browne et al., 2016; Lagier et al., 2016), respiratory tract (Fonkou et al., 2018), genitourinary tract (Thomas-White et al., 2018), oral cavity (Fonkou et al., 2018; Martellacci et al., 2020), and even in other model animals (Lagkouvardos et al., 2016; Liu et al., 2020) mostly adopted this strategy and had isolated hundreds of previously unknown bacteria inhabiting the human body. In addition, live bacteria could facilitate the follow-up mechanism researches regarding their impact on physiological and pathological functions, such as the bile salt (salt ion form of bile acid) metabolism test. Bile acids are cholesterol-derived natural surfactants and are regarded as important digestive hormones that are produced in the liver and secreted into the duodenum via the gallbladder to regulate numerous physiological processes in the host (Harris et al., 2018). They are known to affect lipid digestion, antibacterial defense, glucose metabolism, host metabolism, cancer progression, and innate and adaptive immunity (Shapiro et al., 2018; Hang et al., 2019; Song et al., 2020). The biotransformation of bile acids (BAs) by intestinal bacteria may have an important role in cholesterol gallstone formation and colon carcinogenesis via TGR and FXR (farnesoid-X receptor) receptors (Salvioli et al., 1982; Funabashi et al., 2020). So far, bacteria are mainly involved in two categories of bile acid metabolism. Bile salt hydrolase (BSH) with deconjugation activity hydrolyzes the amide bond that links the bile acid side chain to glycine or taurine and takes part in the transformation process from conjugated bile acid to unconjugated bile acid (Ridlon et al., 2006). Besides, the dehydroxylase activity participates in the transformation process from primary bile acid to secondary bile acid (White et al., 1980). These metabolic activities have mostly been found in intestinal bacteria (Kuipers et al., 2020). However, the ability of bile acid biotransformation by bile bacteria has not been reported to date.

Altogether, the objectives of the study are to (1) cultivate and isolate a large number of bile bacteria of cholecystitis patients and whole-genome sequence parts of isolates, (2) conduct comparative analyses between our isolates and gut isolates in public databases, which revealed different pathways in our isolates, and (3) evaluate the bile salt metabolic capacity of biliary bacteria *in vitro*. This work provided new insights into the bile bacterial communities based on genomes and the biotransformation potentiality of human bile bacteria.

## Materials and Methods

### Sample Collection

Informed consents were obtained from all subjects, and this study was approved by the Medical Ethics Committee of Hunan Provincial People’s Hospital. The study was conducted according to the principles of the Declaration of Helsinki of 1964 and later versions (2013) and was approved by the Medical Ethics Committee of the Hunan Provincial People’s Hospital (Ethical approval number: 2020-54). All the experiments were performed in accordance with relevant guidelines and regulations. The bile samples collected from four postoperative (laparoscopic cholecystectomy) acute cholecystitis patients in Hunan Provincial People’s Hospital were kept in an anaerobic gas bag (Mitsubishi, Japan), respectively, and rapidly transferred to the laboratory for cultivation. To address the potential contamination that might have occurred before and after the samples reached our laboratory, sterile physiological saline control samples without bile were applied in the study.

### Cultivation and Identification of Bile Species

All bile samples were centrifuged at 3,000 × *g* for 5 min and carefully removed most of the supernatant of the bile sample but left around 200 μl of supernatant on the bottom to resuspend the pellet to keep bacteria with the highest density. These bacteria were then spread on different solid media for culture. [BBL—Brain Heart Infusion Agar (Becton Dickinson, United States), *Bifidobacterium* Medium (Becton Dickinson, United States), Lactose Bile (Becton Dickinson, United States), Blood Agar Base Infusion Agar (Becton Dickinson, United States), Columbia Blood Agar Base (Becton Dickinson, United States), BBE Agar (Becton Dickinson, United States), and Gifu Anaerobic Medium (Beijing Land Bridge Technology Co., Ltd, China)] under aerobic and anaerobic conditions at 37°C. Cultivation and identification methods of bile species mainly referred to two studies (Browne et al., 2016; Lagier et al., 2016). Briefly, colonies were picked up and transferred on a fresh medium for the purification of bacterial colony. Isolated colonies were transferred into an 8-ml liquid medium for enrichment culture and were identified using PCR amplification of the 16S rRNA gene (primers: 7F 5′-AGAGTTTGATYMTGGCTCAG-3′; 1510R 5′-ACGGYTACCTTGTTACGACTT-3′) (Browne et al., 2016). The PCR products were applied on Sanger sequencing at ABI 3730XL platform (Applied Biosystems, United States) (Browne et al., 2016). Each 16S rRNA sequence was blasted against the rRNA/ITS databases of the National Center of Biotechnology Information (NCBI) for bacterial strain identification. The identified aerobic strains were preserved in 30% glycerol and anaerobic strains in 30% glycerol and 0.1% cysteine at a −80°C freezer.

### Whole-Genome Shotgun Sequencing, Assembly, and Annotation

The DNA of bacterial isolates was extracted using the Qiagen DNA extraction kit (Qiagen, Germany) according to the protocols of the manufacturer. The DNA concentration and purity were determined by NanoDrop2000. DNA quality was examined with a 1% agarose gel electrophoresis. Bacterial DNA was fragmented to an average size of ∼300 bp using Covaris M220 (Gene Company Limited, China). Paired-end libraries were prepared by using a TruSeq DNA sample prep kit (Illumina, United States). Adapters containing the full complement of sequencing primer hybridization sites were ligated to blunt-end fragments. Paired-end whole-genome shotgun sequencing was performed on the Illumina HiSeq platform. High-quality reads were extracted based on the FASTQ (Chen et al., 2018), with default parameters. High-quality reads were used for *de novo* assembly via SPAdes (Bankevich et al., 2012), using different k-mer sizes (*k* = 21, 33, 55, 77). The shortest scaffolds were filtered with a minimum length threshold of 200 bp. Gene identification was performed from the assembled genome using Prodigal (Hyatt et al., 2010), and the other genomic contents (e.g., rRNA and tRNA sequences) were annotated using the Prokka (Seemann, 2014) pipeline. Protein-coding genes were further annotated to the KEGG (Kyoto Encyclopedia of Genes and Genomes, downloaded on February 2020) (Kanehisa et al., 2017) databases using BLASTP (identity threshold of 35%, covering > 70% of the gene length).

## Identification of Antibiotic Resistance Genes and Virulence Factors

Identification and characterization of antibiotic resistance genes (ARGs) and virulence factors of pathogens are crucial in understanding bacterial pathogenesis and their interactions with the host, and in the development of novel drugs, vaccines, and molecular diagnostic tools. Furthermore, detecting virulence or resistance markers improved outbreak monitoring and therapeutic management. Currently, the use of the next-generation sequencing platforms has allowed great progress in this field (Bakour et al., 2016). ARGs were identified based on ABRicate.^1^ ABRicate searched on the databases, including NCBI Bacterial Antimicrobial Resistance Reference Gene Database, CARD (Jia et al., 2017), ARG-ANNOT (Gupta et al., 2014), and ResFinder (Zankari et al., 2012) for predicting ARGs. The virulence factors of the genome were identified based on the VFDB database (Chen et al., 2016).

### Comparison Genome Analyses

Average nucleotide identity (ANI) between two genomes was calculated using the fastANI algorithm (Jain et al., 2018). An ANI threshold of 95% was used for species delineation for prokaryotic genomes (Jain et al., 2018). The phylogenetic tree of bacterial species was generated using PhyloPhlAn2 (Segata et al., 2013) and visualized using iTOL (Letunic and Bork, 2019).

### *In vitro* Bile Acid Transformations by Bile Bacteria

Bile acids were dissolved in DMSO for preparation of a highly concentrated stock solution. Then, the autoclaved GAM liquid medium was applied to dilute the stock solution (volume ratio of DMSO in a working solution <1‰). The bile acid experimental group [containing 200 ng/ml of final concentration: TCA (taurocholic acid), CA (cholic acid), or GCA (glycocholic acid)] and the control group (without bile acid) were inoculated with isolated bile bacteria in 200 μl of liquid media reaction system at 37°C. A control without bile acid (but with the DMSO vehicle) and a sterile control with an equal volume of bile acid (200 ng/ml) were prepared. Every reaction was prepared in parallel. This experiment was done in triplicates. Bile acids of internal standards were prepared at 200 ng/ml. After 12 h, the cultures were centrifuged (10,000 × *g*, 5 min), and the supernatants were collected. Then, the double volume of acetonitrile was added, and the aqueous phase was collected after centrifuging at 20,000 × *g* for 10 min. The detection was achieved using a Phenomenex Kinetex Polar C18 ODS (2.1 × 100 mm, 2.6 μm) analytical column. The mobile phase consisted of 0.1% ammonium hydroxide water (A) and acetonitrile (B) at a flow rate of 0.3 ml/min, and the following gradient condition was used: 0–1 min 65% A; 1–4 min 65–60% A; 4–5.5 min 60–35% A; 5.5–6.5 min 35–20% A, 6.5–7.0 20–10% A, 7.0–8.0 10% A, 8.0–8.5 10–65% A). An Applied Biosystems AB Sciex Qtrap5500 Mass Spectrometer (MS/MS) equipped with an electron spray ionization (ESI) source was used to analyze target metabolites at negative ionization mode (Scherer et al., 2009). The optimized ion spray voltage and drying gas temperature were set at -4,500 V and 550°C, respectively. The curtain gas (CUR) flow was 35 L/min; gas1 and gas2 (nitrogen) were set at 45 and 55 psi, respectively, and the retention time was 70 ms. Quantification assays were performed using multiple reaction monitoring.

### Statistical Analyses

The value of metabolite output is equal to the output of the experimental group minus the average output of the control group. All statistical analyses were performed based on the R platform. Histograms were performed using GraphPad Prism 7 software (GraphPad Software) and were indicated using the mean with the standard deviation.

## Results

### Cultivation and Genome Sequencing of Bacteria in Bile Specimens

**FIGURE 1:**
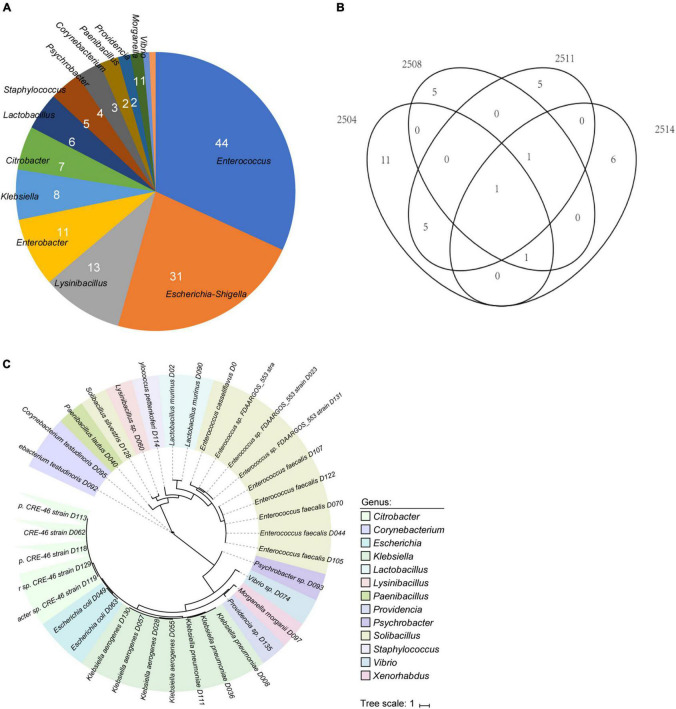
Overview of the cultivated bacteria from bile specimens. **(A)** Pie plot shows the composition of 138 bacterial colonies. **(B)** Venn diagram of the distribution of isolates in the four bile samples. **(C)** Phylogenetic tree of 35 whole-genome sequenced isolates. Color represents the genus assignment of the isolates.

To explore the bile bacterial community, the fresh bile specimens collected from cholecystitis patients were cultivated using seven different media under both aerobic and anaerobic conditions. In total, we obtained 138 bacterial colonies and performed full-length 16S rRNA gene sequencing to enable taxonomic assignment. These isolates included 70 (50.7%) members of Firmicutes, 65 (47.1%) members of Proteobacteria, and 3 (2.2%) members of Actinobacteria; meanwhile, they expanded 3 classes, 6 orders, 10 families, and 14 genera (Supplementary Table 1). At the genus level, *Enterococcus* (31.9%), *Escherichia–Shigella* (22.9%), *Lysinibacillus* (9.4%), and *Enterobacter* (8%) were the most frequent members in the biliary community, while the remaining 10 genera represented 28.2% (39/138) of the cultivated isolates (Figure 1A). *Proteus mirabilis* (24 strains) and *Enterococcus faecalis* (17 strains) were the most abundant species, which may be due to the property of facultative anaerobes (Portela et al., 2014; Jamil et al., 2021). *Enterobacteriaceae* had the largest isolated strains (28 strains) under aerobic conditions (Supplementary Table 1). In addition, 63 strains were obtained under anaerobic conditions (63/138), and most of them were also facultative anaerobes, such as the *Enterococcus* genus.

We clustered the isolates into 35 operational taxonomic units (OTUs) based on 99% nucleotide similarity of their 16S rRNA gene sequences and performed whole-genome sequencing for the representative isolate for each cluster. After *de novo* assembly of the sequencing reads, we obtained 35 high-quality draft genomes that exceeded 95% genomic completeness for each strain (Figure 1B). A phylogenetic tree of the draft genomes was shown in Figure 1C. The genome sizes of these biliary bacterial isolates were on average 4.0 Mbp, ranging from 1.9 to 7.1 Mbp (Supplementary Table 2), and the G + C contents of these genomes ranged from 37.4 to 63.3% (on average, 47.4%). The majority (32/35) of the biliary genomes could be assigned into known species, showing > 95% ANI to at least one sequenced genome in the NCBI database, whereas the other 3 biliary isolates were “novel species” as they previously had no available whole-genome information (Supplementary Table 2). Moreover, despite the 16S rRNA gene sequences of these biliary strains seemed diverse, 26 genomes were further grouped into 8 species-level genome bins based on 95% ANI.

### Characteristics of the Novel Species

**FIGURE 2:**
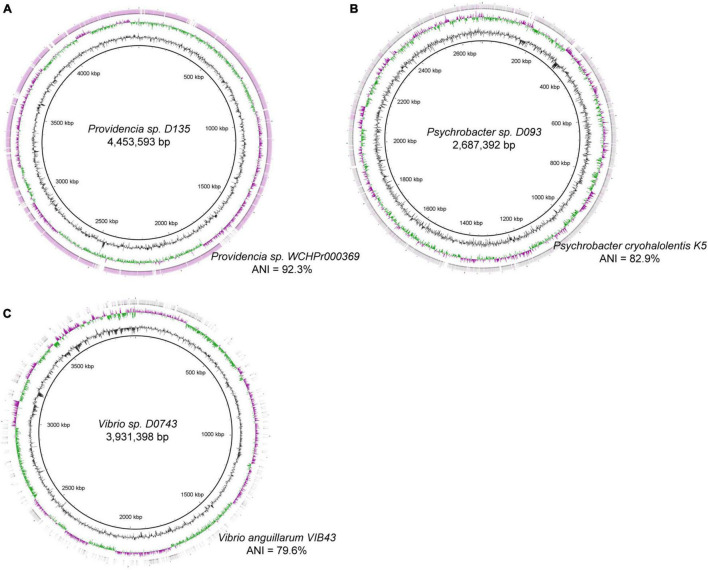
Circular representation of the genomes of four novel species, including **(A)** *Providencia* sp. *D135*, **(B)** *Psychrobacter* sp. *D093*, and **(C)** *Vibrio* sp. *D074*. For each genome, the inner three circles represent the genome scale, G + C skew, and G + C content; the outer circle(s) shows the genome region that have close orthologs with the closest strains in the National Center of Biotechnology Information (NCBI) database. Average nucleotide identity (ANI) of the bile genomes and its closest strains are shown.

We analyzed the genomes of three novel species to investigate the distinctive features of those biliary bacterial species. Biliary strain *Providencia* sp. D135 consisted of 27 contigs with a total length of 4.45 Mbp (N50 length: 315 kbp). This strain was moderate homology with the genome of *Providencia* sp. WCHPr000369, a clinical strain that was isolated from the human rectum (proposed name: *Providencia huaxiensis*) (Hu et al., 2019), with 92.3% ANI (Figure 2A). Biliary strain *Psychrobacter* sp. D093 consisted of 99 contigs with a total length of 2.69 Mbp and showed 82.9% ANI with the genome of *Psychrobacter cryohalolentis* K5 (Figure 2B). Biliary strain *Vibrio* sp. D074 consisted of 87 contigs with a total length of 3.93 Mbp and showed 79.6% ANI with the genome of *Vibrio anguillarum* VIB43 (Figure 2C).

### Comparison of Bile and Gut Isolates

To investigate the specificity of bile bacterial species, we compared our bile isolates with 1,520 bacterial strains cultivated from the gastrointestinal tract of healthy adults (Zou et al., 2019). Based on whole-genome pairwise ANI, 13 strains were specifically detected in bile samples. These bile-specific genomes were mainly distributed in Firmicutes, including *Lactobacillus murinus*, *Enterococcus casseliflavus*, *Paenibacillus lautus*, *Staphylococcus pettenkoferi*, *Lysinibacillus* sp. D060, and *Solibacillus silvestris*, and several Proteobacteria clades, including *Providencia* sp. D135, *Morganella morganii*, *Vibrio* sp. D074, and *Psychrobacter* sp. D093 (Supplementary Figure 1).

**FIGURE 3:**
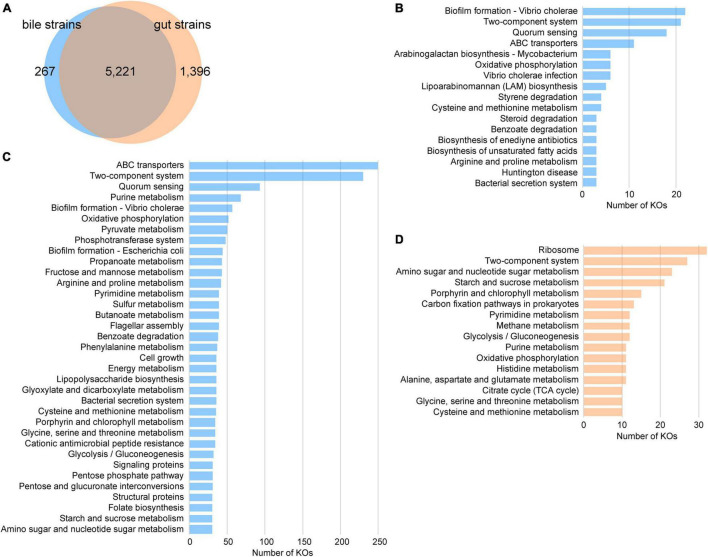
Comparison of the functions of bile and gut bacterial strains. **(A)** Venn diagram shows the comparison of KEGG orthologs (KOs) between bile and gut strains. **(B–D)** Distribution of KEGG level C pathways of the bile-specific KOs **(B)**, the bile-enriched KOs **(C)**, and the gut-enriched KOs **(D)**.

The functional roles of the members of bile and gut strains were compared based on their profiles of the KEGG orthologs (KOs). The bile strains encoded 5,488 KOs, of which 95.1% were also encoded by the gut strains (Figure 3A). The bile-specific KOs were involved in pathways including biofilm formation, two-component systems, quorum sensing, and ABC transporters (Figure 3B). We also undertook an enrichment analysis and identified 3,740 statistically enriched KOs and 695 reduced KOs in bile bacteria (Benjamini–Hochberg-adjusted *p* < 0.05, Fisher’s exact test). The bile-enriched KOs had frequently performed some key functions, including ABC transporters, two-component system, quorum sensing, purine metabolism, biofilm formation, oxidative phosphorylation, pyruvate metabolism, and phosphotransferase system, while the bile-reduced KOs were involved in functions such as the ribosome, two-component system, amino sugar and nucleotide sugar metabolism, and starch and sucrose metabolism (Figures 3C,D).

### Antibiotic Resistance Genes, Virulence Factors, and Bile Acid Metabolism Genes

**FIGURE 4:**
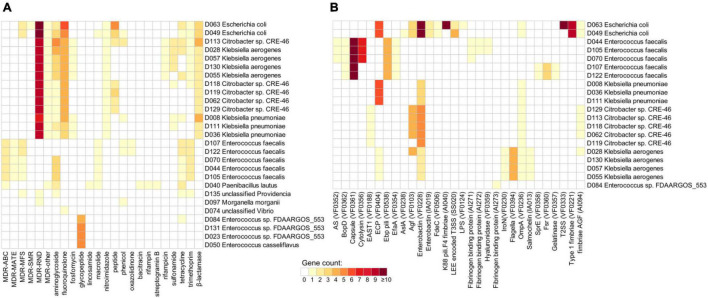
Distribution of antibiotic resistance genes (ARGs) and virulence factors in the bile bacterial strains. Heat maps show the number of ARGs **(A)** and virulence factors **(B)** for each strain.

Next, we examined the existence of ARGs and virulence factors in the genomes of bile species, aiding to extend their potential clinical pathologic features. A total of 472 genes from 27 genomes were identified as ARGs based on annotations in the available antibiotic resistance databases (Supplementary Table 3). Most of the ARGs were related to multidrug resistance (MDR) (42.6%), fluoroquinolone resistance (12.3%), aminoglycoside resistance (9.1%), and β-lactamase (7.2%). The remaining genes were involved in resistance against various types of antibiotics such as tetracycline (4.4%), glycopeptide (4%), peptide (4%), and trimethoprim (4%). At the species level, *Escherichia coli*, *Citrobacter* sp. CRE-46, *Klebsiella aerogenes*, and *Klebsiella pneumoniae* encoded a higher number of ARGs than other bile species, especially that their ARGs were enriched in types including MDR, fluoroquinolone resistance, and β-lactamase (Figure 4A). *Enterococcus faecalis* frequently encoded the genes involving trimethoprim, macrolide, and tetracycline resistances, while *Enterococcus casseliflavus* and *Enterococcus* sp. FDAARGOS_553 encoded the genes of glycopeptide resistance. In addition, we identified 354 genes involving virulence factors from 20 bile strains (Supplementary Table 4). The virulence factors were mainly distributed in species including *Escherichia coli*, *Enterococcus faecalis*, *Klebsiella pneumoniae*, *Citrobacter* sp. CRE-46, and *Klebsiella aerogenes* (Figure 4B). In addition, we identified a total of 12 genes encoding the BSH and 319 genes involved in the transformation of primary bile acids to secondary bile acids (i.e., the multiple steps 7α/β-dehydroxylation) (Supplementary Table 5). These genes spanned all 35 genome-sequenced strains, suggesting that the bile bacteria have widely participated in bile acid metabolism. At the species level, *Lysinibacillus* sp. D060, *Solibacillus silvestris*, *Paenibacillus lautus*, and the members of *Klebsiella* (i.e., *K. aerogenes* and *K. pneumoniae*) had the largest number of bile acid metabolism genes.

### Evaluation of Bile Salt Metabolism Potentiality of Bile Bacteria

**FIGURE 5:**
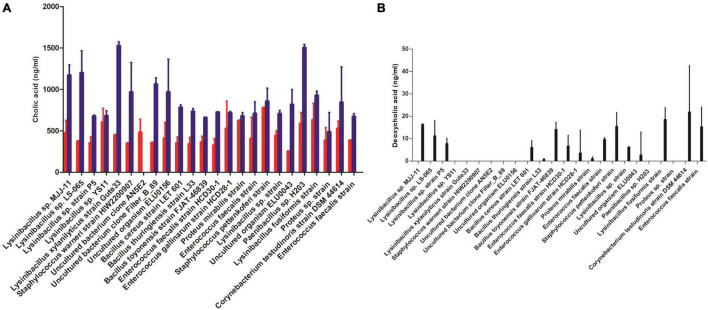
Detection of bile salt metabolism by bile bacterial strains. The concentration of metabolites **(A)** converted glycocholic acid (GCA) to cholic acid (CA) (red bar), or taurocholic acid (TCA) to CA (blue bar) **(B)** converted CA to deoxycholic acid (DCA).

We evaluated the potential BSH and dehydroxylase activities in 24 strains, which were selected by phylogenetic tree based on 16S rRNA gene amplicon sequence by measuring the changed concentration of free bile acids or secondary bile acids in the supernatant of cultures. *Paenibacillus* sp. H203 (Ps) and *Lysinibacillus xylanilyticus* Gute33 (Lx) were detected with strong bile salt deconjugation capabilities. Cholic acid formed with GCA substrate [586.1 ± 135 ng/ml (Ps), 445.4 ± 14.1 ng/ml (Lx)] and 1,500.6 ± 42 ng/ml (Ps), 1,525.4 ± 49 ng/ml (Lx) cholic acid formed with TCA substrate (Figure 5A). *Lysinibacillus* sp. MJJ-11 and *Corynebacterium testudinoris* DSM 44614 strain showed the stronger capabilities of forming secondary bile acid, which converted CA to DCA (Figure 5B).

## Discussion

The bile bacteria, including the *Vibrio* genus especially *Vibrio cholerae* (Asnis et al., 1996), *Escherichia coli*, *Klebsiella* spp., *Enterococcus* (Yun and Seo, 2018), *Staphylococcus*, *Corynebacterium* (Backert et al., 2018), and *Lactobacillus salivarius*, have been studied extensively (Woo et al., 2002). Tajeddin et al. (2016) found that enterobacteria were the dominant biliary bacteria based on cultivate-dependent methods. As an alternative animal model of humans, most of the bile and gall bladder bacteria of healthy pigs were *Streptococcus alactolyticus*, *Staphylococcus epidermidis*, and *Corynebacterium testudinoris*, which suggested that healthy host bile may also contain bacteria (Jimenez et al., 2014). In our previous research and the work of others, the bile pathogenic bacteria were considered a predisposing factor in gallbladder disorders (Liu et al., 2015; Shen et al., 2015; Tajeddin et al., 2016). Furthermore, metagenomic results indicated that many potential bile bacteria still cannot be isolated and cultivated (Liu et al., 2015). With more cultivated and detected conditions termed “culturomics” emerging (Lagier et al., 2016), many previously unavailable “dark matter bacteria” from bile would be isolated.

In this study, we characterized the isolated bacterial genome from the fresh bile specimens from four cholecystitis patients and evaluated its bile acid biotransformation ability *in vitro*. Our results showed that the number of cultivable bile bacteria exceeds expectations, spanning 138 bacterial isolates that were distributed in 14 genera. In particular, three bacterial species (i.e., *Providencia* sp. D135, *Psychrobacter* sp. D093, and *Vibrio* sp. D074) were not represented in existing reference genome databases. Enterobacteriaceae had the most isolated strains (28 strains) under aerobic conditions, in agreement with previous studies (Tajeddin et al., 2016). Although there was little research on bile anaerobes, we obtained 63 strains under anaerobic conditions, however, most of them were facultative anaerobes. The possible reasons were the small sample size (*n* = 4) and the nutrient composition of the medium, which was not specifically optimized or not rich enough for bile bacteria. For instance, Lagier et al. (2016) reported that liquid media precultivation operation can effectively promote the diversity of isolated bacteria. In addition, the centrifugal operation may lose some lighter bacteria; therefore, after centrifugation, the supernatant can be aseptically filtered (Spin-X Centrifuge Tube Filter, Corning Inc.) and concentrated, and the concentrated part will be used for cultivation to obtain more bacteria. The isolated bacterial genomes could extend the databases, which are the basis for metagenomics analysis (Lagier et al., 2016).

Whole-genome sequencing was performed on 35 bacterial species and compared with gut isolates. Our results showed that many pathways, such as biofilm formation, ABC transporters, and quorum sensing, were enriched in bile bacteria, which may contribute to communicate with each other and resist the antibacterial high concentration of bile salts (Davidson et al., 2008; Moons et al., 2009; Rutherford and Bassler, 2012). Bile salts promoted the biofilm formation of bile bacteria, and the biofilms of bacteria-laden gallstones influenced the illness severity (Stewart et al., 2007). Especially, the biofilm formation ability seems to be a necessary condition for bile resistance and polymicrobial infection in this organ (Tajeddin et al., 2016; Zheng et al., 2017). Therefore, this high abundance of functional pathways of bile bacteria, such as biofilm formation, was related to its living environment and physiological characteristics.

Enterobacteriaceae had the largest isolated strains (28 strains) under aerobic conditions in this study which was consistent with the previous studies (Yun and Seo, 2018) and had been identified to be closely related to cholecystitis in our previous research (Liu et al., 2015). ARGs and virulence factors analyses of the bacterial isolates further revealed that important Gram-negative opportunistic pathogen of the Enterobacteriaceae, such as *Escherichia coli*, *Citrobacter* sp. CRE-46, *Klebsiella aerogenes*, and *Klebsiella pneumoniae*, encoded the higher number of ARGs and the virulence factors than other bile species. This result was inconsistent with those of previous studies that less than 5% resistance was observed in bile isolated bacteria against carbapenem, beta-lactam antibiotics, glycopeptide antibiotics, and linezolid via the antibiotic susceptibility tests (Yun and Seo, 2018). This difference may be caused by different test methods and their sensitivity. Therefore, the combination of the bioinformatics and the verification of conventional drug-susceptibility testing method on the pivotal isolates in the next step may have positive significance in the quicker selection of the most suitable antibiotics for clinical applications.

In humans, the major bile salts included taurocholic acid (TCA) and glycocholic acid (GCA) derived from cholic acid (CA), while taurochenodeoxycholic acid (TCDCA) and glycochenodeoxycholic acid (GCDCA) derived from chenodeoxycholic acid (CDCA). The salts of their 7-alpha-dehydroxylated derivatives, deoxycholic acid (DCA), and lithocholic acid (LCA), are also found, with derivatives of cholic, chenodeoxycholic, and deoxycholic acids accounting for over 90% of human biliary bile acids (Hofmann, 1999). Bile acids are closely related to human health and can be metabolized by gut bacteria, which generated free bile acid by deconjugation and generated secondary bile acids by 7α-and 7β-dehydroxylation, such as deoxycholic acid and lithocholic acid (Ridlon et al., 2016; Wang and Jia, 2016; Marion et al., 2019; Song et al., 2019; Funabashi et al., 2020). Secondary bile acids, especially DCA, have been listed as carcinogens and promoters of CRC (Yang and Yu, 2018). High-fat diet-induced high levels of DCA in the intestine are also considered an important risk factor for CRC. Herein, based on the phylogenetic tree of isolates, we selected 24 bile bacterial strains and detected their bile salt deconjugation and transformation abilities. *Lysinibacillus* genus had strong bile acid biotransformation ability (Figure 5), which was consistent with previous reports that *Paenibacillus* and *Lysinibacillus* widely carried BSH (Song et al., 2019). This ability of bile bacteria may allow them to overcome the bile salt damaging effect. Although this study provided direct evidence *in vitro* for the bile bacteria involved in the biotransformation of bile acid, however, we are not sure that the bacteria-mediated transformations occur in the gallbladder or not so far. The reason is that the presence of *in vivo* bile acid transformations performed by certain isolated microorganisms lacks rigor since the same strain may display completely different functional performance between its *in vivo* and *in vitro* activity (Marion et al., 2019). For instance, *Clostridium hiranonis* did not deconjugate tauro-conjugated bile acids in germ-free mice, although its ability to deconjugate taurocholic acid (TCA) was shown *in vitro* (Narushima et al., 1999). Importantly, our research results provided new insights for screening bile salt metabolizing bacteria, which many studies focused on gut bacteria (Lucas et al., 2021).

Our data helps in understanding the genetic basis of the physiology, biochemical pathways, and evolution of the isolates and provides a preliminary exploration in bile acid transformations by bile bacteria; however, the sample size and culture conditions were relatively small. Therefore, larger-scale bacterial isolation and identification, more in-depth screening isolates with bile acid transformation ability *in vivo* by metabolite detection method, and verifying the impact of metabolite on gallbladder and even whole-body health are to be investigated in the next step.

## Data Availability Statement

The datasets presented in this study can be found in online repositories. The names of the repository/repositories and accession number(s) can be found below: https://www.ncbi.nlm.nih.gov/bioproject/PRJNA726166, PRJNA726166.

## Ethics Statement

The studies involving human participants were reviewed and approved by the Medical Ethics Committee of the Hunan Provincial People’s Hospital (Ethical approval number: 2020-54). The patients/participants provided their written informed consent to participate in this study.

## Author Contributions

YM, QY, SL, YX, and XHM contributed to conception and design of the study. SL, SZ, YZ, QL, and AZ organized the formal analysis. GW, RL, MX, TJ, YS, XL, SC, XT, and XD performed methodology. YM, XCM, CW, and YX were responsible for project administration. QY, SL, and SZ wrote the first draft of the manuscript. All authors were involved in preparing the manuscript and contributed to manuscript revision, read, and approved the submitted version.

## Conflict of Interest

The authors declare that the research was conducted in the absence of any commercial or financial relationships that could be construed as a potential conflict of interest.

## Publisher’s Note

All claims expressed in this article are solely those of the authors and do not necessarily represent those of their affiliated organizations, or those of the publisher, the editors and the reviewers. Any product that may be evaluated in this article, or claim that may be made by its manufacturer, is not guaranteed or endorsed by the publisher.

## Footnotes

1
https://github.com/tseemann/abricate

## Supplementary Material

Supplementary Figure 1 Phylogenetic tree of 35 bile isolates and 1,520 bacterial strains cultivated from the feces. Outer color bars represent the phylum assignment of the isolates.
