# Supplementary figures and images for: Cultivation and Genomic Characterization of the Bile Bacterial Species From Cholecystitis Patients
Source: Front Microbiol. 2021 Nov 1;12:739621. doi: 10.3389/fmicb.2021.739621 (PMC8591784; doi:10.3389/fmicb.2021.739621)

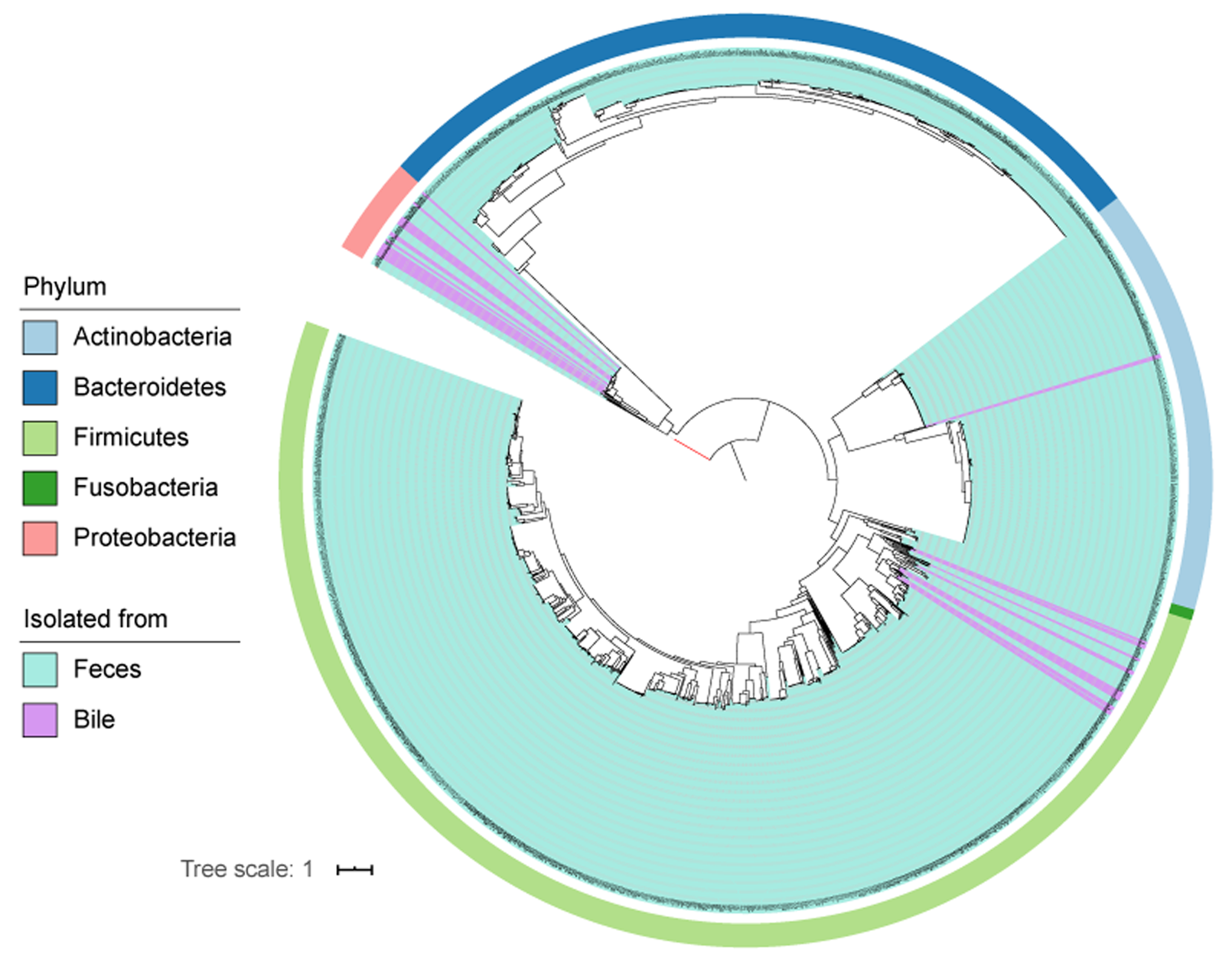

Supplement: Supplementary Figure 1 — Phylogenetic tree of 35 bile isolates and 1,520 bacterial strains cultivated from the feces. Outer color bars represent the phylum assignment of the isolates. [file Image_1.TIF]
